# FOXA1 and AR in invasive breast cancer: new findings on their co-expression and impact on prognosis in ER-positive patients

**DOI:** 10.1186/s12885-018-4624-y

**Published:** 2018-07-03

**Authors:** Nelson Rangel, Nicoletta Fortunati, Simona Osella-Abate, Laura Annaratone, Claudio Isella, Maria Graziella Catalano, Letizia Rinella, Jasna Metovic, Renzo Boldorini, Davide Balmativola, Pietro Ferrando, Francesca Marano, Paola Cassoni, Anna Sapino, Isabella Castellano

**Affiliations:** 1 Department of Medical Sciences, University of Turin, Via Santena 7, 10126 Turin, Italy; 2 Natural and Mathematical Sciences Faculty, University of the Rosario, Bogotá, Colombia; 3 Oncological Endocrinology Unit, Città della Salute e della Scienza Hospital, Turin, Italy; 4 Candiolo Cancer Institute – FPO, IRCCS, Candiolo, Italy; 5 Division of Pathology, Department of Health Sciences, University of Eastern Piedmont and Maggiore Hospital, Novara, Italy; 6 Division of Breast Surgery, Department of General and Specialized Surgery, Città della Salute e della Scienza Hospital, Turin, Italy

**Keywords:** Breast cancer, Prognosis, FOXA1, Androgen receptor, Immunohistochemistry, Real-time PCR

## Abstract

**Background:**

The role of forkhead-box A1 (FOXA1) and Androgen receptor (AR) in breast cancer (BC) has been extensively studied. However, the prognostic role of their co-expression in Estrogen receptor positive (ER+) BC has not been investigated so far. The aim of the present study was thus to assess the co-expression (protein and mRNA) of FOXA1 and AR in BC patients, in order to evaluate their prognostic impact according to ER status.

**Methods:**

Immunohistochemical expression of AR and FOXA1 was evaluated on 479 consecutive BC, with complete clinical-pathological and follow up data. Fresh-frozen tissues from 65 cases were available. The expression of AR and FOXA1 with ER was validated using mRNA analyses. Survival and Cox proportional hazard analyses were used to evaluate the relationship between FOXA1, AR and prognosis.

**Results:**

Expression of ER, AR and FOXA1 was observed in 78, 60 and 85% of cases respectively. Most AR+ cases (97%) were also FOXA1+. The level of FOXA1 mRNA positively correlated with level of both AR mRNA (*r* = 0.8975; *P* < 0.001) and ER mRNA (*r* = 0.7326; *P* < 0.001). In ER+ BC, FOXA1 was associated with a good prognosis independently of AR expression in the three subgroups analyzed (FOXA1+/AR+; FOXA1+/AR-; FOXA1−/AR-). Multivariate analyses confirmed that FOXA1 may provide more information than AR in Disease-Free Interval (DFI) of ER+ BC patients.

**Conclusion:**

Our results suggest that in BC the expression of FOXA1 is directly related to the expression of AR. Despite that, FOXA1 is found as superior predicting marker of recurrences compared to AR in ER+ BC patients.

**Electronic supplementary material:**

The online version of this article (10.1186/s12885-018-4624-y) contains supplementary material, which is available to authorized users.

## Background

In breast cancer (BC), Estrogen (ER) and Androgen Receptors (AR) regulate cell proliferation and differentiation. They are frequently co-expressed, however AR may be expressed in ER-negative (ER-) BC, where it modulates gene transcription by using regulatory molecules and pathways normally activated by ER [1]. As a result, in ER- BC cells, androgens activate cell proliferation [2], whereas in ER-positive (ER+) cells, androgens inhibit cell proliferation [3, 4]. In line with these data, we demonstrated that patients with AR+/ER+ BC have a better prognosis compared to those affected by AR−/ER+ BC [5, 6].

FOXA1, a member of the forkhead family protein [7], is an important regulator of ER DNA binding and transcription of its target genes [8]. In addition, in both ER+ and ER- BC cells, FOXA1 promotes AR DNA binding [1, 9, 10]. Several studies [11–19] evaluated the prognostic role of FOXA1 in BC, and demonstrated that in ER+ BC the expression of FOXA1 is positively correlated with a better prognosis. Indeed, the role of FOXA1/AR co-expression in ER+ BC has not been investigated, although it has been suggested that the relative ratio among FOXA1, ER and AR could influence growth and aggressiveness of cancer cells [20].

The aim of the present study was first to assess the co-expression, at both protein and mRNA levels, of FOXA1 and AR in BC, and then to evaluate their prognostic impact in ER+ BC patients.

## Methods

### Case series

We collected a series of 479 female patients that underwent surgery for BC from June 1994 to December 2012 at the Breast Unit of the Città della Salute e della Scienza Hospital of Turin, Italy. All patients were treated with surgery, either mastectomy or wide local excision, followed by radiotherapy.

Clinical-pathological data such as age at time of diagnosis, surgery (conserving surgery vs radical mastectomy), type of therapy (hormonal therapy, chemotherapy), type and site of recurrences, histological types, tumor size (< 15 mm vs ≥ 15 mm), nodal involvement, histologic grade and vascular invasion were collected. Medical charts of all patients were reviewed to confirm accuracy of previously recorded data. Tumor slides were re-evaluated to select representative blocks that were used to construct multicore tissue microarrays (TMAs, tissue arrayer Galileo TMA CK 3500, Integrated Systems Engineering Srl, Milan, Italy), as previously described [21].

### Immunohistochemistry

To confirm the results of the diagnostic reports, immunohistochemistry (IHC) was performed on TMA sections using an automated slide processing platform (Ventana BenchMark AutoStainer, Ventana Medical Systems, Tucson, AZ, USA) and the following primary antibodies were used: prediluted anti-ER rabbit monoclonal antibody (SP1, Ventana-Roche, Tucson, AZ, USA); prediluted anti-Progesterone receptor (PgR) rabbit monoclonal antibody (1E2, Ventana-Roche); anti-Ki67 monoclonal antibody (MIB1, diluted 1:100 Dako); anti-human c-erbB2 oncoprotein (Ventana Pathway HER-2/Neu-4B5). In addition, AR and FOXA1 expression were tested using anti-AR mouse monoclonal antibody (AR441, diluted 1:50, Dako, Glostrup, Denmark) and prediluted anti-FOXA1 mouse monoclonal antibody (2F83, Ventana-Roche). Positive and negative controls (omission of the primary antibody and IgG-matched serum) were included for each IHC run.

The cut-off value for ER and PgR expression was set at 1%, as suggested by St Gallen Consensus meeting [22], and the same cut-off was also adopted for AR and FOXA1 expression [5]. The percentage of Ki67-positive cells was recorded and the cut-off for dichotomizing tumors with low and high proliferative fraction was established at 20% according to 2013 St Gallen Consensus meeting [23] and also on the basis of the median Ki67 value of our local laboratory [24, 25]. HER2 status was classified as negative (score 0, 1+ and 2+ not amplified) or positive (when scored 3+ by IHC or HER2 amplified by FISH) according to the recommended guidelines for invasive carcinoma [26].

### Real-time PCR (qPCR) analysis

To determine the specificity of AR and FOXA1 antibodies, we compared gene expression levels (using qPCR) with IHC results. The relationship between AR and FOXA1 was validated using relative quantification mRNA analyses.

**Fig. 1:**
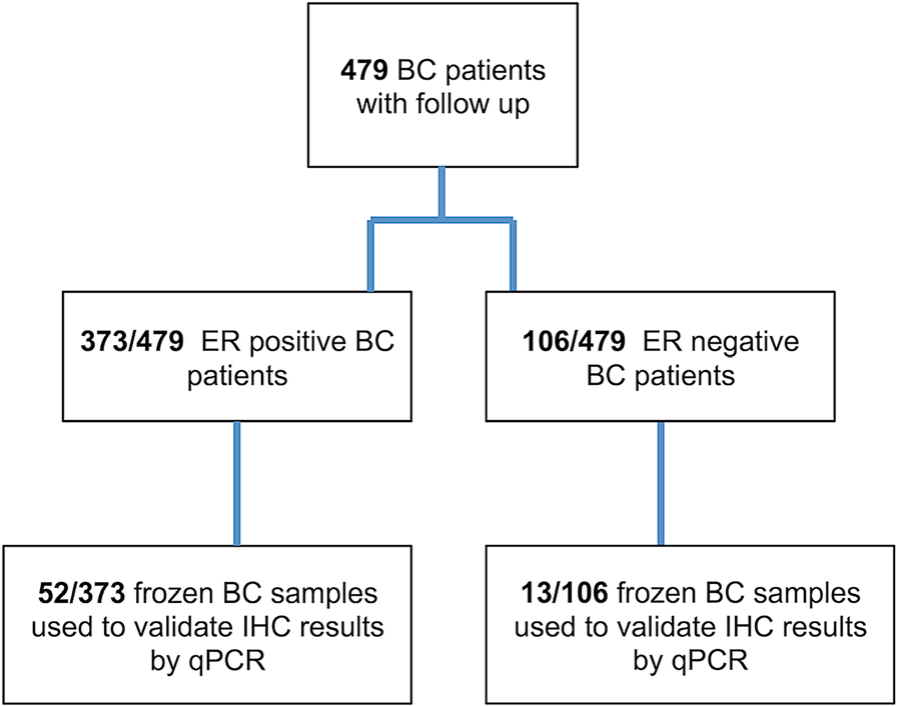
Flow chart of the study

qPCR for AR and FOXA1 mRNA was performed on 65 fresh-frozen BC samples (Fig. 1). Total RNA was extracted from tissues using TRIzol Reagent (Invitrogen Ltd., Paisley, UK) following manufacturer’s instructions. DNase I was added to remove remaining genomic DNA. 1 μg of total RNA was reverse-transcribed with iScript cDNA Synthesis Kit (Bio-Rad Laboratories Inc., Hercules, CA, USA), following manufacturer protocol. Primers (Additional file 1: Table S1) were designed using Beacon Designer 5.0 software according to parameters outlined in the Bio-Rad iCycler Manual. Specificity of primers was confirmed by BLAST analysis. qPCR was performed using a BioRad iQ iCycler Detection System (Bio-Rad Laboratories Inc., Hercules, CA, USA) with SYBR green fluorophore. Reactions were performed in a total volume of 25 μl containing 12.5 μl of IQ SYBR Green Supermix (Bio-Rad Laboratories Inc., Hercules, CA, USA), 1 μl of each primer at 10 μM concentration, and 5 μl of the previously reverse-transcribed cDNA template. The protocol used was as follows: denaturation (95 °C for 5 min) and amplification repeated 40 times (95 °C for 15 s, 60 °C for 30 s). At each run, a melting curve analysis was performed to ensure a single specific amplified product for every reaction. Results were normalized using the Delta-Ct (Δct) method, using β-actin as housekeeping gene. Samples with a Δct ≤ 6 were defined as positive.

### Statistical and survival analyses

Pearson’s Chi square test and Student’s t-test were preliminary performed to compare respectively categorical and continuous variables, and to evaluate potential differences in the variable distribution among groups. Test for median and means (Analysis of Variance-ANOVA) were performed. For more than two groups Tukey HSD post-hoc test was performed. Disease-Free Interval (DFI) was calculated from the date of surgical excision of the primary tumor to the date of first disease relapse or last check-up. Disease-specific survival (DSS) was calculated from the surgical excision date of the primary tumor to the date of BC death or last check-up [24, 27]. Survival distribution curves were plotted using the Kaplan-Meier method and the statistical comparisons were performed using the log-rank test. Cox regression analyses were carried out on DFI and DSS to calculate crude and adjusted HRs and 95% confidence intervals (CIs) for the different study group. Cases lost to follow up and cases with a non-BC related cause of death were censored at the last follow up control. The step-wise model selection method was used to determine the final Cox regression model. Akaike Information Criterion test (AIC) and likelihood ratio test (LRT) were carried out to measure how selected variables improve parsimony and goodness of fit of the selected model. The proportional hazard assumption was assessed with the Schoenfeld residuals. This did not give reasons to suspect violation of this assumption. All statistical tests were two sided. *P*-values < 0.05 were considered significant. Statistical analyses were performed using Stata/SE12.0 Statistical Software (STATA, College Station, TX).

## Results

### Association of FOXA1 and AR IHC expression with clinical-pathological characteristics

Clinical and histopathological characteristics of the whole population are reported in Table 1. The median follow up was 10.1 years (7,7-12,7). The majority of patients was over 50 years (> 80%) of age and underwent conservative surgery. Positive expression of ER, AR and FOXA1 was observed in 78, 60 and 85% of cases respectively. As previously reported [13, 15], in our cohort FOXA1 positivity was associated with small tumor size (< 15 mm), absence of lymph node metastases, low histological grade, no special type (NST) histotype, low level of Ki67, as well as, with ER+ and PgR+ tumors (Additional file 1: Table S2). In the consecutive series of patients, 58% of cases showed AR+/FOXA1+ (Table 2), while 14% presented AR-/FOXA1- immunophenotype and only 1.7% of cases were AR+/FOXA1-. This latter subgroup, did not show specific features. Compared to the other subgroups, FOXA1-/AR- BC phenotype was more frequently associated with high histological grade, large tumor size, no expression of ER and PgR and high proliferation index (*P* < 0.001) (Additional file 1: Table S3).

**Table 1:** Clinical and histopathological characteristics of 479 breast cancer patients

Characteristics	N (%)
Age	
≤ 50	86 (18)
> 50	393 (82)
Type of surgery (missing 8 cases)	
Conservative	282 (59.9)
Mastectomy	189 (40.1)
Size (missing 7 cases)	
< 15 mm	176 (36.7)
≥15 mm	296 (63.3)
Lymph node involvement (missing 7 cases)	
pN0	277 (58.7)
pN1–3	195 (41.3)
Histological Grade (missing 9 cases)	
1	125 (26.6)
2	187 (39.8)
3	158 (33.6)
Histotype	
NST - CDI	305 (63.7)
CLI	95 (19.8)
others	79 (16.5)
Vascular invasion (missing 113 cases)	
No	200 (54.6)
Yes	166 (45.4)
ER	
0	106 (22.1)
≥ 1%	373 (77.9)
PR (missing 48 cases)	
0	122 (28.3)
≥ 1%	309 (71.7)
Ki67 (missing 9 cases)	
< 20%	205 (43.6)
≥20%	265 (56.4)
HER2 (missing 43 cases)	
Negative	398 (91.3)
Positive	38 (8.7)
FOXA1	
Negative	74 (15.4)
Positive	405 (84.6)
AR	
Negative	193 (40%)
Positive	286 (60%)
Therapy (missing 15)	
Only radiotherapy	18 (3.9%)
Hormonal therapy	229 (49.4%)
Chemo-Hormonal	125 (26.9%)
Chemotherapy	81 (17.4%)
No therapy	11 (2.4%)
Recurrences	
No	389 (76.9%)
Yes	90 (23.1%)
Deaths	
No	440 (91.9%)
Yes	39 (8.1%)

**Table 2:** Association between FOXA1 expression and AR status according to immunohistochemistry test

	FOXA1 positive	FOXA1 negative	*P* Value*
AR Positive	278	8	<0.001
AR Negative	127	66	

* Chi-Square (X^2^)

### qPCR analysis: Correlation between mRNA and protein levels of FOXA1 and AR in BC

**Fig. 2:**
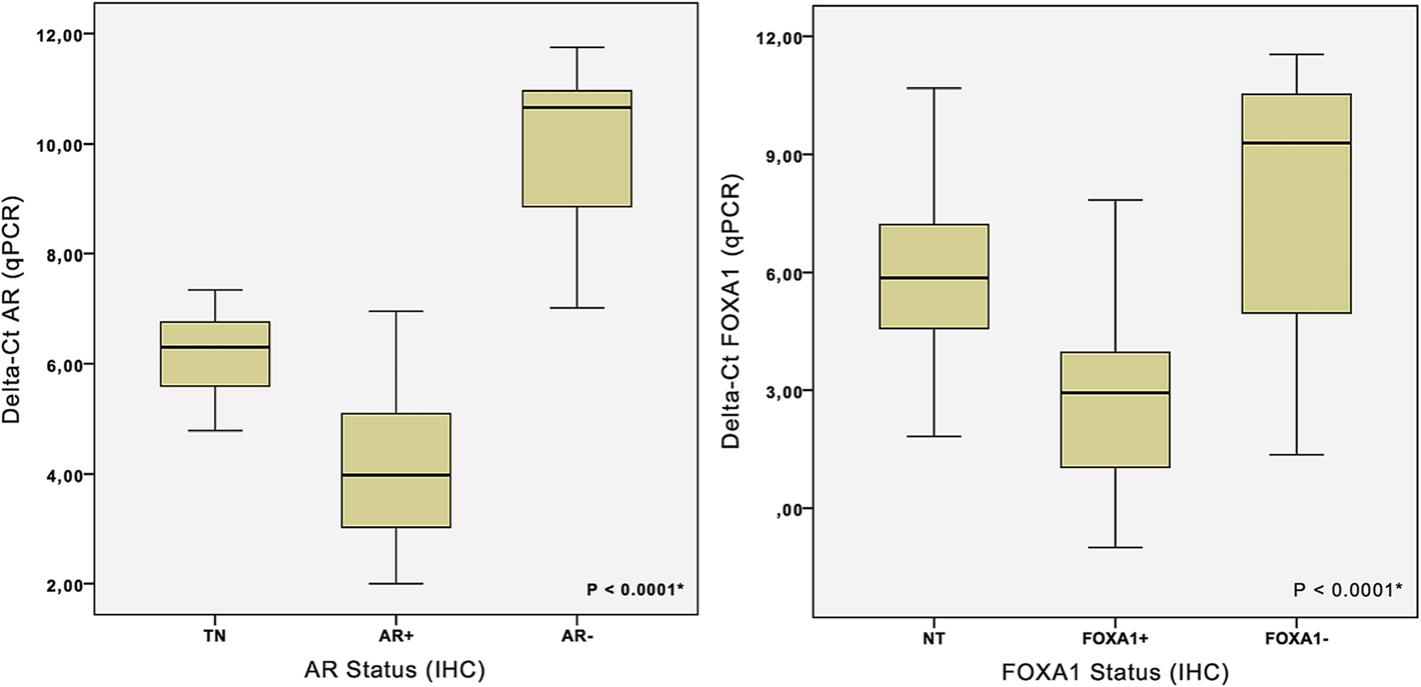
Protein (IHC) and mRNA (qPCR) expression for Androgen receptor (AR) and Forkhead box protein A1 (FOXA1). It can be observed that positive protein expression (AR and FOXA1) correlates with higher mRNA levels (low delta-Ct). Tukey’s multiple comparisons test showed significant differences between positive and negative cases, for both AR and FOXA1 (*p* < 0.0001). NT - Normal Tissue. *ANOVA analysis

**Fig. 3:**
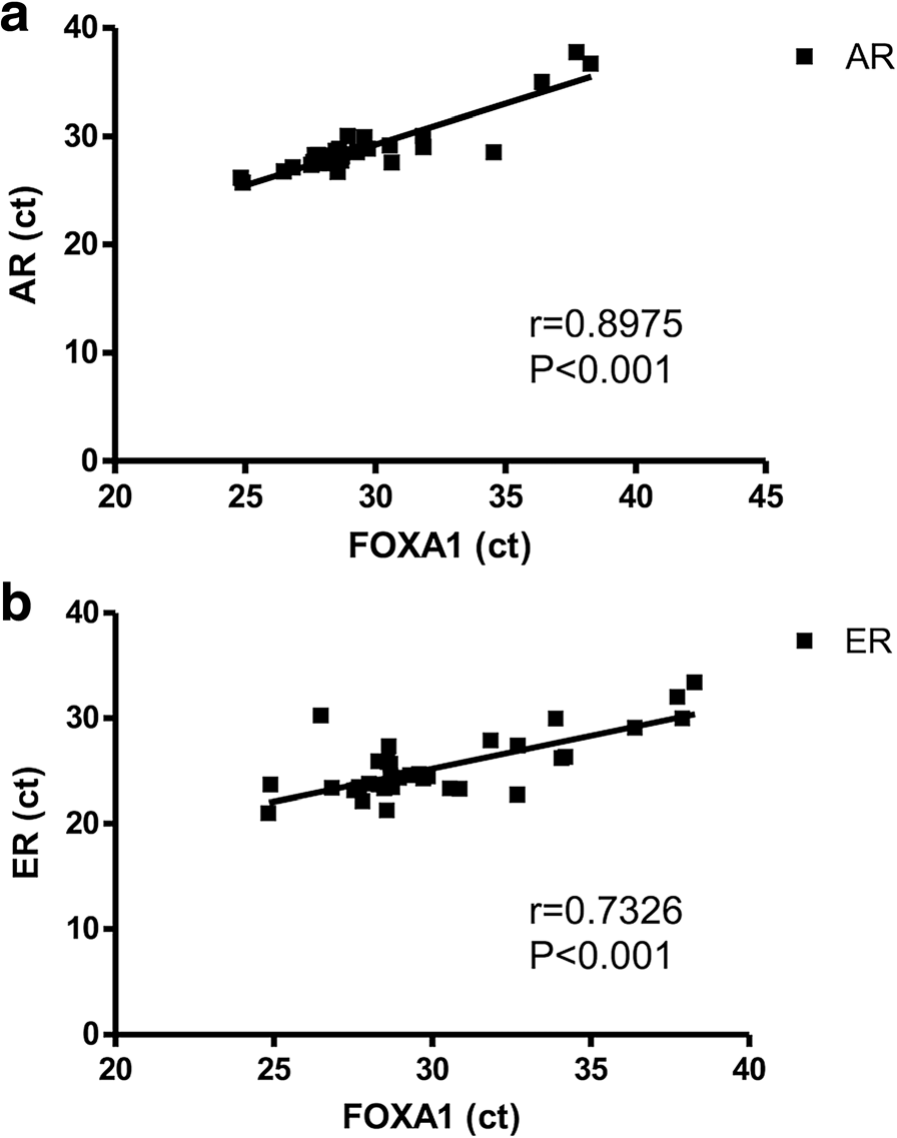
Spearman’s correlation test, show that FOXA1 mRNA level positively correlated with mRNA levels of **a**. Androgen receptor (AR) and **b**. Estrogen receptor (ER)

We found a strict correlation of FOXA1 and AR mRNA and protein expression (Fig. 2). To correlate the expression of ER, AR and FOXA1, we decided to use qPCR results, because this procedure allows quantifying more precisely the level of expression of each molecule. As shown in Fig. 3, there was a linear correlation (Spearman’s correlation) of the level of FOXA1 mRNA with the level of AR (*r* = 0.8975; *P* < 0.001) (Fig. 3a) and ER (*r* = 0.7326; *P* < 0.001) mRNA (Fig. 3b).

**Fig. 4:**
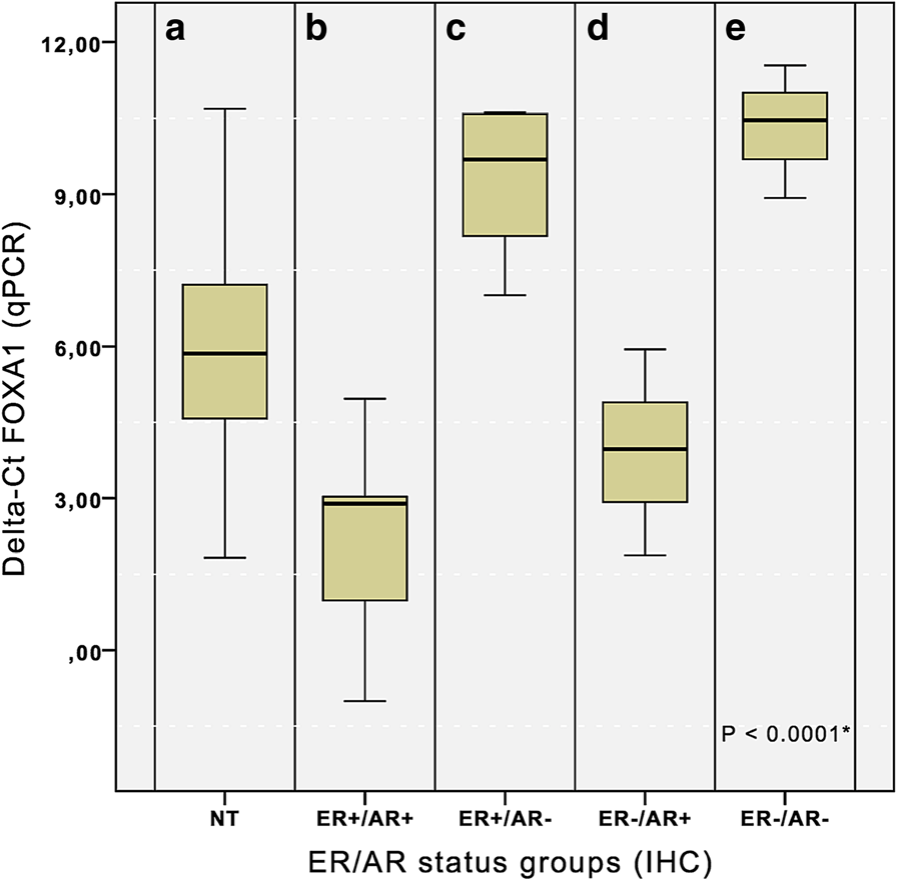
FOXA1 mRNA expression in: **a**. NT - Normal Tissue; **b**. ER+/AR+ tumors; **c**. ER+/AR- tumors; **d**. ER−/AR+ tumors; **e**. ER−/AR- tumors. Independently of ER status, FOXA1 mRNA levels were higher (low delta-Ct) in AR+ tumors, compared to AR- cases. Tukey’s multiple comparisons test showed significant differences, mainly, between groups with AR+ and AR- cases (*p* < 0.0001. Additional file 1: Table S4). *ANOVA analysis

Furthermore, FOXA1 mRNA was closely related to AR mRNA expression, regardless of ER status. Indeed, FOXA1 mRNA was expressed in all samples with ER+/AR+ (27 cases) and ER−/AR+ (3 cases) (Low delta-Ct. Fig. 4b and d), in 8/25 ER+/AR- cases and in only 1/10 ER−/AR- cases (High Delta-Ct. Fig. 4c and e).

### Impact of FOXA1 and AR IHC co-expression on prognosis

At univariate analysis performed on whole cohort, metastatic lymph nodes, histological grade, vascular invasion, ER and PR positivity, high Ki67 and HER2 overexpression were confirmed as significant prognostic factors. Additionally, the expression of AR and FOXA1 were associated with a better DFI and DSS (Table 3, Additional file 1: Figure S1).

**Table 3:** Univariate analysis

Characteristics	DFI			DSS		
	HR	CI	P	HR	CI	P
Age	0.98	0.96–1.00	0.110	1.00	0.98–1.03	0.727
Conservative vs Mastectomy	3.36	2.22–5.10	0.000	2.56	1.42–4.63	0.002
Lymph node involvement						
0	1			1		
1	2.01	1.19–3.42	0.009	1.27	0.54–2.97	0.581
2	5.63	3.22–9.86	0.000	5.26	2.44–11.3	0.000
3	12.1	6.74–21.7	0.000	12.5	5.61–27.7	0.000
Histotype						
NST - CDI	1			1		
CLI	0.75	0.44–1.26	0.275	1.03	0.52–2.06	0.925
Other	0.59	0.30–1.15	0.122	0.40	0.12–1.32	0.132
Hystological grade						
1	1			1		
2	2.53	1.35–4.74	0.004	3.34	1.12–9.94	0.030
3	4.31	2.30–8.05	0.000	7.52	2.61–21.7	<0.001
Tumor Size > 15 mm	4.98	2.46–10.1	0.000	5.42	1.90–15.4	0.002
Vascular invasion	5.16	2.99–8.90	0.000	3.84	1.86–7.93	0.000
ER Positive	0.44	0.28–0.69	0.000	0.34	0.18–0.64	0.001
PGR > 20%	0.63	0.41–0.97	0.034	0.37	0.19–0.69	0.002
Ki67 ≥ 20%	3.10	1.96–4.92	0.000	4.15	1.99–8.64	0.000
FOXA1 Positive	0.54	0.32–0.90	0.019	0.43	0.21–0.88	0.022
AR positive	0.60	0.40–0.91	0.015	0.38	0.21–0.70	0.002
HER2 Positive	2.50	1.25–5.00	0.010	1.98	0.70–5.58	0.195

Clinical and pathological data correlated with disease free interval (DFI) and disease specific survival (DSS)

**Fig. 5:**
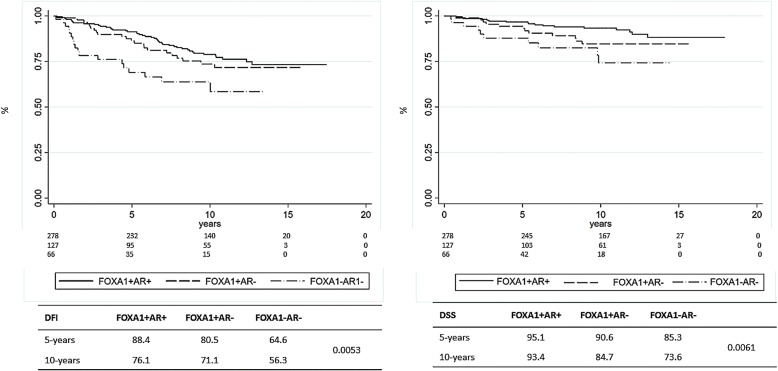
Kaplan–Meier estimates of DFI and DSS according to AR and FOXA1 in all breast tumors

To analyze the impact of FOXA1 and AR in patients with BC (ER+ or ER-), we created three BC subgroups (FOXA1+/AR+; FOXA1+/AR-; FOXA1−/AR-). We were unable to perform any analyses on the FOXA1−/AR+ BC since only 8 patients carried this phenotype (Table 2). As shown in Fig. 5, in the consecutive series of patients, the lack of expression of both, FOXA1 and AR (FOXA1−/AR-), was related to a worse DFI and DSS compared to the other groups.

**Fig. 6:**
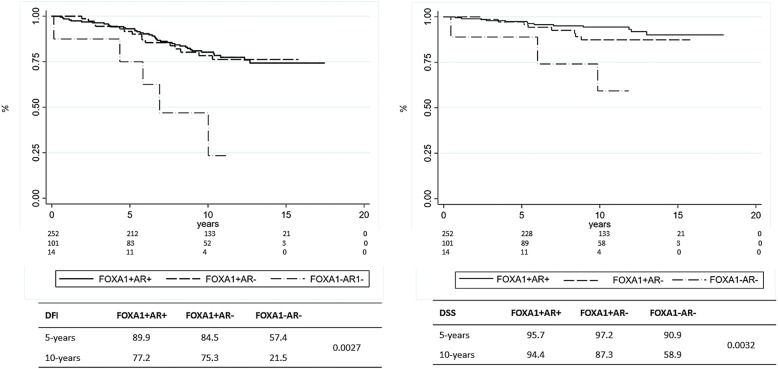
Kaplan–Meier estimates of DFI and DSS according to AR and FOXA1 in ER+ breast cancer patients

Finally, we investigated the relationship between FOXA1, AR and prognosis in BC patients stratified for ER expression. As shown in Fig. 6, in ER+ BC, FOXA1 expression was closely related to good prognosis independently of AR expression.

Multivariate analyses (Table 4) performed on ER+ BC confirmed that FOXA1 may provide more information than AR on DFI, but not on DSS. In the subset of patients with ER- BC, FOXA1, alone or in association with AR, did not show any relationship with outcome (data not shown).

**Table 4:** Multivariate analysis

Characteristics	DFI (global test *p* = 0.5497)^a^			DSS (global test *p* = 0.7496)^a^		
	HR	CI	P	HR	CI	P
Age	0.99	0.97–1.02	0.703	0.99	0.96–1.04	0.844
Size ≥ 15 mm	2.40	1.00–5.78	0.050	5.40	0.65–44.6	0.117
Lymph node involvement						
0	1					
1	2.01	0.89–4.52	0.090	1.23	0.27–5.54	0.790
2	4.24	1.68–10.7	0.002	4.27	1.07–17.0	0.040
3	6.34	2.24–17.9	0.001	6.58	1.45–29.9	0.015
KI67 ≥ 20%	2.58	1.19–5.58	0.016	4.27	0.88–20.7	0.071
FOXA1	0.24	0.08–0.74	0.013	0.34	0.04–3.08	0.340

Association of tumor characteristics with disease free interval (DFI) and disease specific survival (DSS) among ER+ patients with complete data for all covariates

^a^Test of proportional-hazards assumption

## Discussion

We assessed, for the first time, the expression of FOXA1 and AR in BC, evaluating their prognostic impact according to ER status. We found that (i) the expression (protein and mRNA) of FOXA1 and AR was closely related: the majority of cases expressing AR showed FOXA1 positivity, conversely, negative expression of FOXA1 correlates with very low level of AR; (ii) the expression of FOXA1 is strictly related to good outcome, and in the subgroup of patients with ER+ BC may provide more information on DFI than AR.

FOXA1 is a “winged helix” transcription factor. It was demonstrated that, by interacting with histones H3 and H4, FOXA1 is responsible for opening compacted chromatin [28], permitting efficient interaction of ER with its response elements. For this reason, the presence of FOXA1 suggests a functional ER complex, which probably will respond to endocrine therapy [11, 29, 30]. Moreover, FOXA1 seems to have a repressor effect on BC growth by promoting transcription of E-cadherin and cell cycle-dependent kinase inhibitor p27(Kip1), thus reducing the motility and invasion of BC cells [31, 32]. These findings suggest that FOXA1 expression in BC may be associated with a better clinical outcome. In our study we confirmed literature data, demonstrating that FOXA1 is mainly expressed in low grade, lymph node negative BC tumors, with size < 15 mm and low Ki67 index [15, 33, 34].

In addition, FOXA1 has been associated with recruitment of AR [7] and, it has been suggested that in prostate epithelium FOXA1 acts with AR in promoting differentiation [35].

ChIP-seq analysis of AR, ER, and FOXA1 in BC cell lines revealed a significant level of co-occupancy between these markers, presumably due to the presence of forkhead motif found at AR and ER binding sites [8, 10, 36, 37]. Furthermore, evidences of the relationship between AR and FOXA1 was supported by experiments demonstrating the co-localization of the two proteins on chromatin [1, 9, 37]. Our results support the evidence of those studies, showing that BC tumor with high mRNA level of FOXA1 are generally ER and AR enriched. On the contrary, tissues with low FOXA1 mRNA level present low level of hormonal receptors, especially of AR.

In several studies has been demonstrated that AR expression is a favorable prognostic marker of disease outcome in ER+ BC [5, 6]. This result has recently been confirmed in a meta-analysis conducted on 17,000 women with early-stage breast cancer [38]. The present work confirms the prognostic role of AR. However, the concurrent evaluation of the expression of both AR and FOXA1, shows that FOXA1 is superior to AR as prognostic marker in patients with BC, especially in ER+ cases. In fact, FOXA1 expression was always related to a better outcome even if AR was not detectable. Similar results were recently obtained in prostate cancer [39], in which it has been demonstrated that FOXA1 expression is closely related to prognosis independently of AR level. Hence, in FOXA1+ BC patients, similar results regarding prognosis were found in AR- and AR+ cases. Thus, we suppose that in ER+ BC patients, FOXA1 could be more important than AR as a marker of better prognosis. Actually, several studies suggested that functionality of AR as well as ER may depend on FOXA1 activity [1, 8, 11].

Sahu, B et al. suggested that in prostate cancers FOXA1 level may contribute to select specific AR binding sites on DNA, activating different gene expression signatures [39]. In our case series we observed very low number of AR+/FOXA1- cases; moreover, as shown in Fig. 4, the expression of these markers seems to correlate. Therefore, we hypothesize that FOXA1 in ER+ BC may control the level of AR expression.

## Conclusions

Our results suggest that in BC the expression of FOXA1 is directly proportional to the expression of AR. Despite that, FOXA1 is found as a superior predicting marker of recurrences compared to AR in ER+ BC patients. Therefore, FOXA1 expression evaluated by IHC on ER+ BC specimens could be considered in routine diagnosis as an additional support to oncologists in the definition of the patient prognosis.

## Additional file

Additional file 1: **Table S1.** Primers for real-time PCR. **Table S2.** Patients’ clinical and histopathological characteristics according to FOXA1 expression. **Table S3.** Clinical and histopathological characteristics of BC patients according to FOXA1 and AR status. **Table S4.** Multiple comparisons of FOXA1 mRNA expression in tumors classified according to ER and AR status. **Figure S1.** Kaplan–Meier estimates of a) disease free interval and b) disease-specific survival according to FOXA1 status in 479 breast tumors. (DOCX 662 kb)

## Abbreviations

AIC: Akaike information criterion
AR: Androgen receptor
DFI: Disease-free interval
DSS: Disease-specific survival
ER: Estrogen receptor
FOXA1: Forkhead-box A1 protein
HER2: Human epidermal growth factor receptor 2
PgR: Progesterone receptor
qPCR: Real-time PCR
