# Supplementary material for: FOXA1 and AR in invasive breast cancer: new findings on their co-expression and impact on prognosis in ER-positive patients
Source: BMC Cancer. 2018 Jul 3;18:703. doi: 10.1186/s12885-018-4624-y (PMC6029370; doi:10.1186/s12885-018-4624-y)

**Additional file 1**

**Table S1** Primers for real-time PCR

| ERα | Sense: 5’-TGT GTC CAG CCA CCA ACC AG- 3’  Antisense: 5’- TTC AAC ATT CTC CCT CCT CTT CGG-3’ |
| --- | --- |
| FOXA1 | Sense: 5’-GGG TGG CTC CAG GAT GTT AGG - 3’  Antisense: 5’-GGG TCA TGT TGC CGC TCG TAG -3’ |
| AR | Sense: 5’-AAT TGT CCA TCT TGT CGT CTT CGG - 3’  Antisense: 5’-GCC TCT CCT TCC TCC TGT AGT TTC -3’ |
| β-ACT | Sense: 5’-GCG AGA AGA TGA CCC AGA TC- 3’  Antisense: 5’- GGA TAG CAC AGC CTG GAT AG-3’ |
| β2-microglobulin | Sense: 5’-AGA TGA GTA TGC CTG CCG TGT G-3’  Antisense: 5’-TCA ACC CTC CAT GAT GCT GCT TAC-3’ |
| L13A | Sense: 5’-GCA AGC GGA TGA ACA CCA ACC-3’  Antisense: 5’-TTG AGG GCA GCA GGA ACC AC-3’ |

**Table S2** Patients’ clinical and histopathological characteristics according to FOXA1 expression

| **Characteristics** | | **FOXA1 Positive**  **N=405 (85%)** | **FOXA1 Negative**  **N=74 (15%)** | ***P*** |
| --- | --- | --- | --- | --- |
| *Age* | <50 | 65 | 21 | 0.011 |
|  | >50 | 340 | 53 |  |
| *Type of surgery*  *(missing 8)* | Conservative | 238 | 44 | 0.0062 |
|  | Mastectomy | 159 | 30 |  |
| *Size (missing 7)* | <15 mm | 162 | 14 | 0.002 |
|  | ≥15 mm | 237 | 59 |  |
| *Lymph node involvement*  *(missing 7)* | pN0 | 234 | 43 | 0.002 |
|  | pN1,2,3 | 165 | 30 |  |
| *Histological grade (missing 9)* | 1 | 118 | 7 | <0.001 |
|  | 2 | 175 | 12 |  |
|  | 3 | 104 | 54 |  |
| *Histotype* | IDC | 256 | 49 | 0.005 |
|  | ILC | 89 | 6 |  |
|  | Other | 60 | 19 |  |
| *Vascular Invasion*  *(missing 113)* | No | 172 | 28 | 0.448 |
|  | Yes | 138 | 28 |  |
| *ER (IHC)* | 0 | 52 | 54 | <0.001 |
|  | >1 | 353 | 20 |  |
| *PgR (IHC)*  *(missing 48)* | 0 | 75 | 47 | <0.001 |
|  | >1 | 288 | 21 |  |
| *Ki67*  *(missing 9)* | <20 | 196 | 9 | <0.001 |
|  | ≥20 | 202 | 63 |  |
| HER2  (missing 43) | Neg | 333 | 65 | 0.611 |
|  | Pos | 33 | 5 |  |

**Table S3** Clinical and histopathological characteristics of BC patients according to FOXA1 and AR status

| **Characteristics** | | **Total** | **FOXA1+AR+** | **FOXA1+AR-** | **FOXA1-AR-** | ***P**** | **FOXA1-AR+** |
| --- | --- | --- | --- | --- | --- | --- | --- |
| *Age* | <50 | 86 | 48 | 17 | 21 | 0.006 | 0 |
|  | >50 | 393 | 230 | 110 | 45 |  | 8 |
| *Type of surgery*  *(missing 8)* | Conservative | 282 | 158 | 80 | 40 | 0.533 | 4 |
|  | Mastectomy | 189 | 114 | 45 | 26 |  | 4 |
| *Size*  *(missing 7)* | <15 mm | 176 | 126 | 36 | 10 | <0.001 | 4 |
|  | ≥15 mm | 296 | 146 | 91 | 55 |  | 4 |
| *LN involvement*  *(missing 7)* | pN0 | 277 | 164 | 70 | 38 | 0.696 | 5 |
|  | pN1,2,3 | 195 | 109 | 56 | 27 |  | 3 |
| *Histological grade (missing 9)* | 1 | 125 | 82 | 36 | 5 | <0.001 | 2 |
|  | 2 | 187 | 127 | 48 | 8 |  | 4 |
|  | 3 | 158 | 60 | 44 | 52 |  | 2 |
| *Histotype* | NST | 305 | 165 | 91 | 46 | <0.001 | 3 |
|  | ILC | 95 | 72 | 17 | 4 |  | 2 |
|  | Other | 79 | 37 | 23 | 16 |  | 3 |
| *VI*  *(missing 113)* | No | 200 | 121 | 51 | 22 | 0.361 | 6 |
|  | Yes | 166 | 92 | 46 | 26 |  | 2 |
| *ER (IHC)* | 0 | 106 | 26 | 26 | 52 | <0.001 | 2 |
|  | >1 | 373 | 252 | 101 | 14 |  | 6 |
| *PgR (IHC)*  *(missing 48)* | 0 | 122 | 49 | 26 | 43 | <0.001 | 4 |
|  | >1 | 309 | 193 | 95 | 19 |  | 2 |
| *Ki67*  *(missing 9)* | <20 | 205 | 144 | 52 | 6 | <0.001 | 3 |
|  | ≥20 | 265 | 126 | 76 | 60 |  | 3 |
| *HER2*  *(missing 43)* | Neg | 398 | 231 | 102 | 57 | 0.105 | 8 |
|  | Pos | 38 | 17 | 16 | 5 |  | 0 |
| *Therapy*  *(missing 15)* | RT | 18 | 4 | 4 | 8 | <0.001 | 2 |
|  | HT | 229 | 157 | 63 | 8 |  | 1 |
|  | CT+HT | 125 | 85 | 32 | 7 |  | 1 |
|  | CT | 81 | 22 | 14 | 41 |  | 4 |
|  | No Therapy | 11 | 6 | 3 | 2 |  | 0 |
| *Recurrences* | No | 389 | 232 | 104 | 46 | 0.036 | 7 |
|  | Yes | 90 | 46 | 23 | 20 |  | 1 |
| *Deaths* | No | 440 | 261 | 115 | 56 | 0.049 | 8 |
|  | Yes | 39 | 17 | 12 | 10 |  | 0 |

* We were unable to perform any analyses on the FOXA1-/AR+ BC since only 8 patients carried this phenotype, therefore P value corresponds to analyses performed between the other three subgroups.

LN: Lymph Node; VI: Vascular Invasion; NST: infiltrative carcinoma of non special type; CLI: infiltrative lobular carcinoma; RT: radiotherapy; HT: Hormonal Therapy; CT: Chemotherapy.

**Table S4** Multiple comparisons of FOXA1 mRNA expression in tumors classified according to ER and AR status

| **Group comparisons** | **Adjusted P-Value*** |
| --- | --- |
| NT vs. ER+/AR+ | <0,0001 |
| NT vs. ER+/AR- | 0,0185 |
| NT vs. ER-/AR+ | 0,1804 |
| NT vs. ER-/AR- | 0,0132 |
| ER+/AR+ vs. ER+/AR- | <0,0001 |
| ER+/AR+ vs. ER-/AR+ | 0,2619 |
| ER+/AR+ vs. ER-/AR- | <0,0001 |
| ER+/AR- vs. ER-/AR+ | 0,0001 |
| ER+/AR- vs. ER-/AR- | 0,9413 |
| ER-/AR+ vs. ER-/AR- | 0,0002 |

***** Tukey’s multiple comparisons test.

**Figure S1** Kaplan–Meier estimates of a) disease free interval and b) disease-specific survival according to FOXA1 status in 479 breast tumors.

b)

a)

252 212 133 21 0 101 83 52 3 0


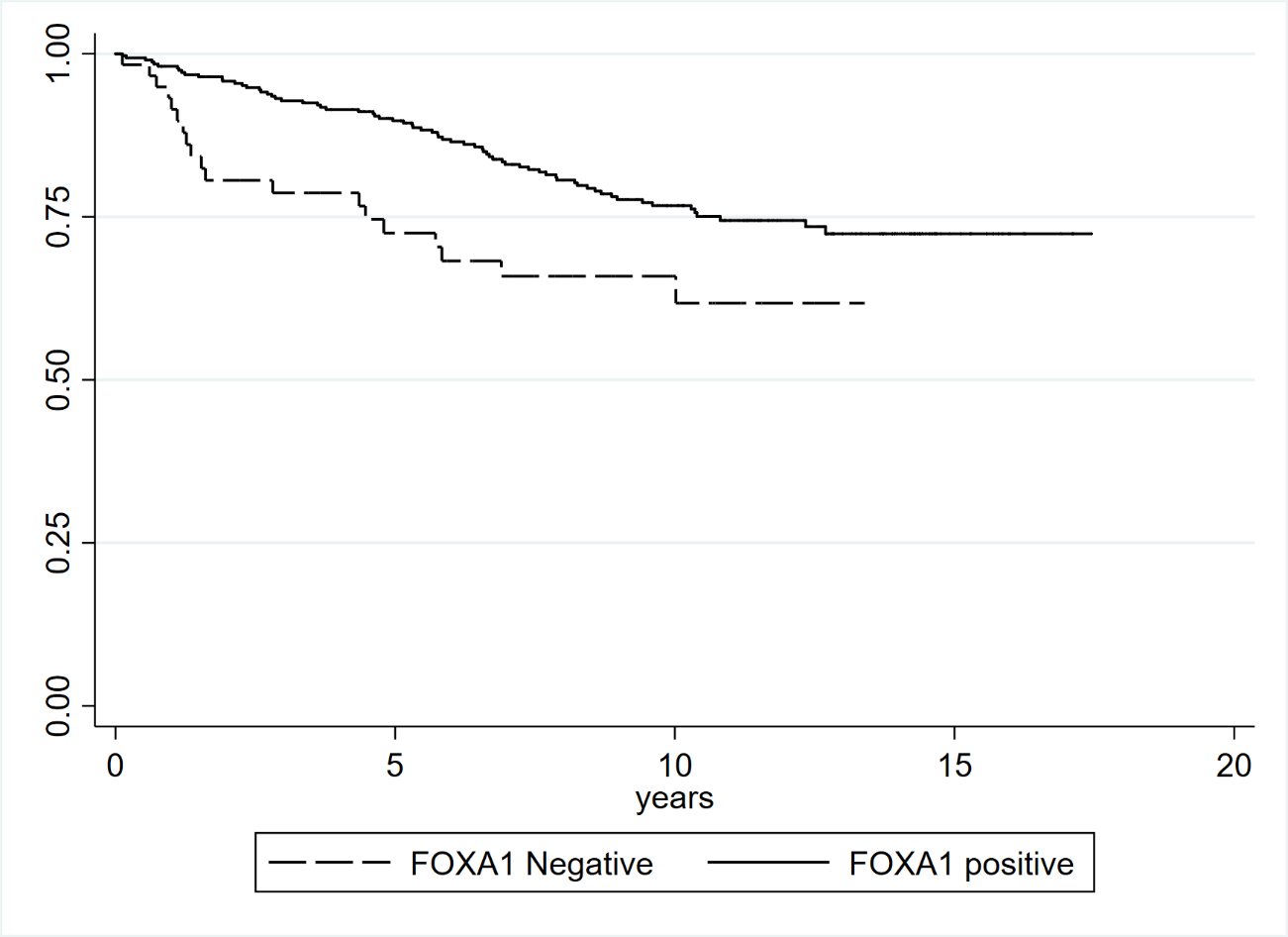


**a)**

252 212 133 21 0

101 83 52 3 0


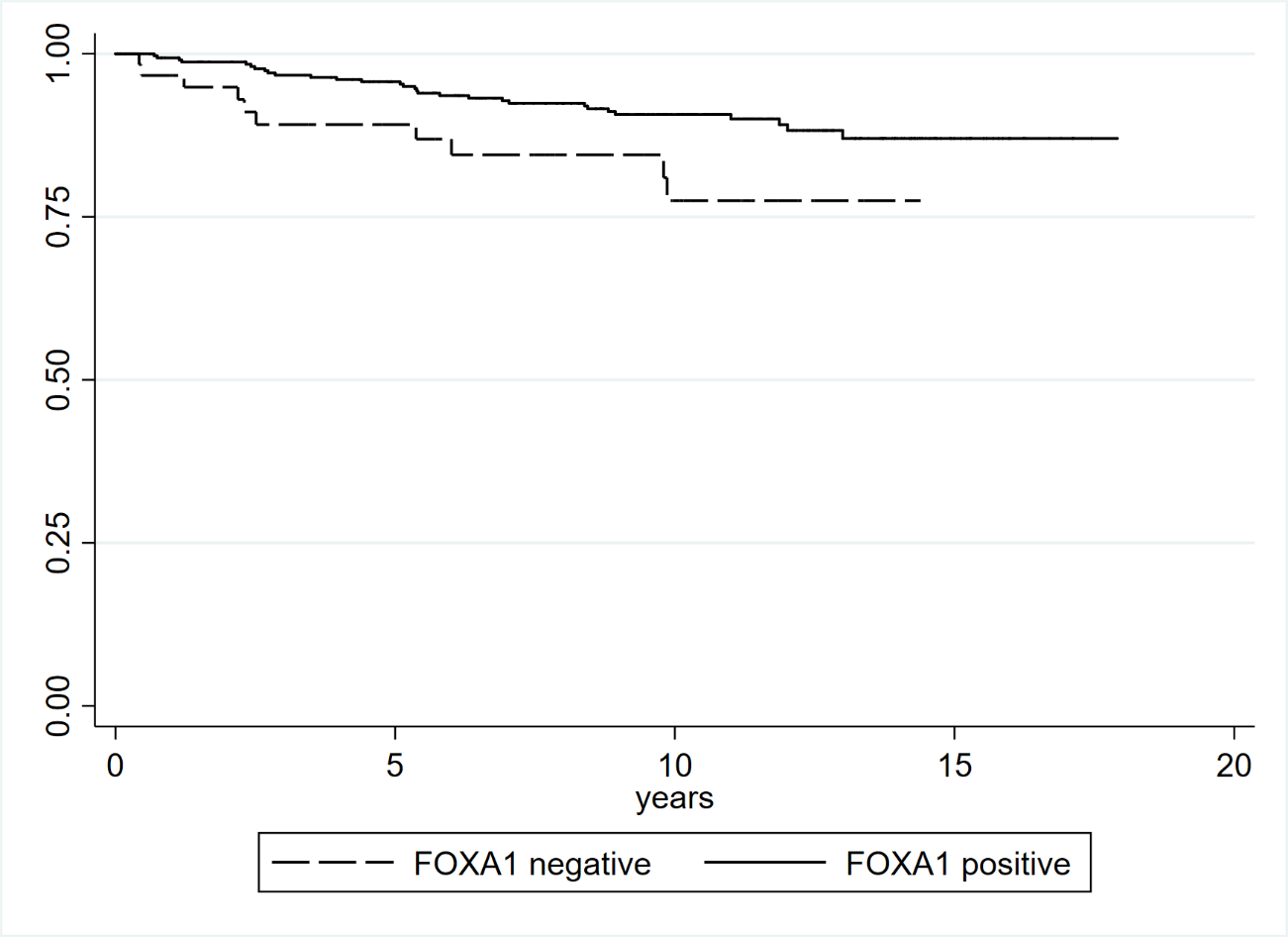


**b)**


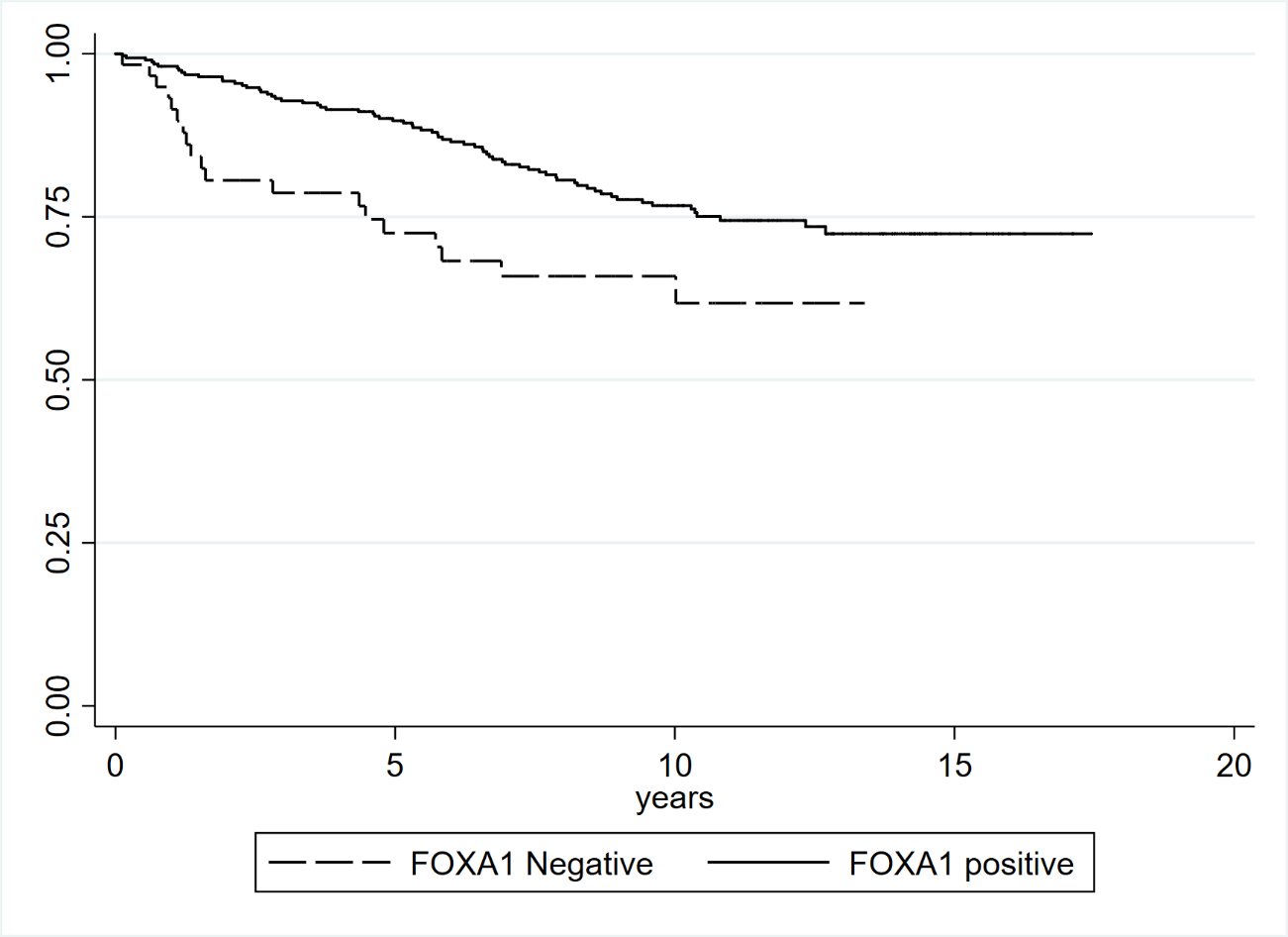

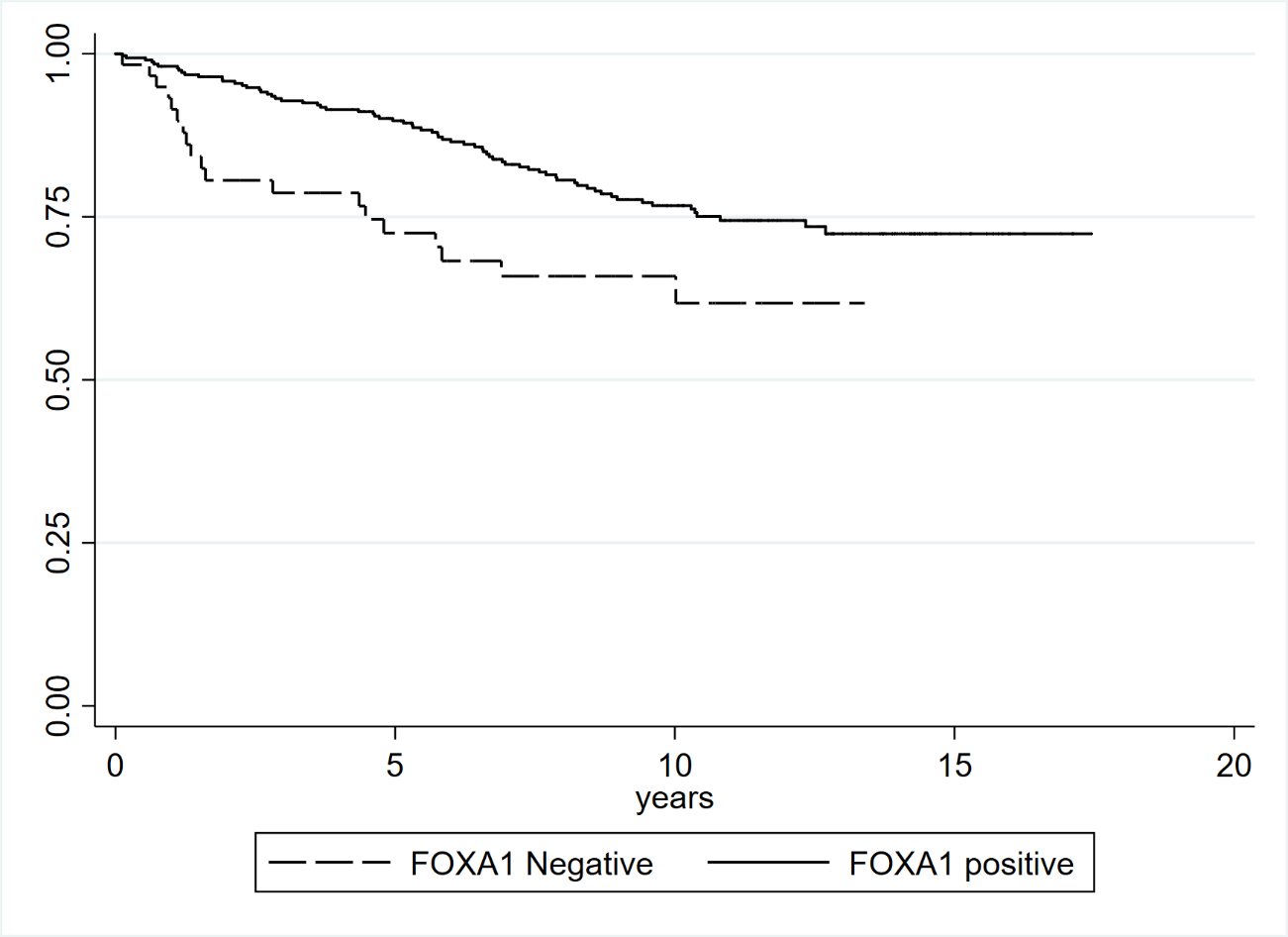


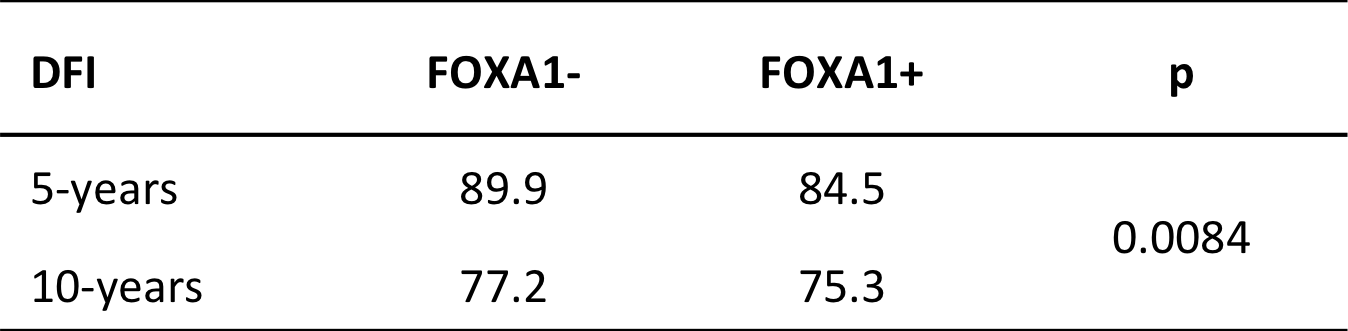

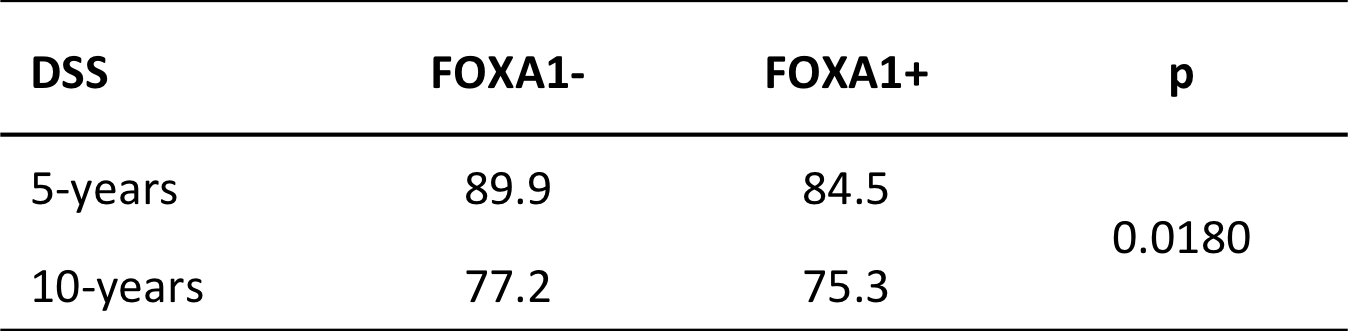

Supplement: Supplementary file 1 — Table S1. Primers for real-time PCR. Table S2. Patients’ clinical and histopathological characteristics according to FOXA1 expression. Table S3. Clinical and histopathological characteristics of BC patients according to FOXA1 and AR status. Table S4. Multiple comparisons of FOXA1 mRNA expression in tumors classified according to ER and AR status. Figure S1. Kaplan–Meier estimates of a) disease free interval and b) disease-specific survival according to FOXA1 status in 479 breast tumors. (DOCX 662 kb) [file 12885_2018_4624_MOESM1_ESM.docx]
